# Caring for young minds: general practitioners’ self-assessed competence in child and adolescent psychiatry

**DOI:** 10.1080/02813432.2026.2654175

**Published:** 2026-04-15

**Authors:** Lars Söderström, Rajna Knez, Sofia Dalemo

**Affiliations:** a Healthcare centre Centrum Praktikertjänst AB, Skövde, Sweden; b Research, Education, Development and Innovation, Primary Health Care, Region Västra Götaland, Sweden; c The Skaraborg Institute, Skövde, Sweden; d Gillbergcentrum, Institute of Neuroscience and Physiology, Sahlgrenska Academy, University of Gothenburg, Göteborg, Sweden; e Child and Adolescent Medicine, Child and Adolescent Psychiatry and Women’s Health, Skaraborg Hospital, Skövde, Sweden; f School of Health Sciences, University of Skövde, Sweden; g Närhälsan Guldvingen Healthcare Centre, Lidköping, Sweden; h Public Health and Community Medicine, Institute of Medicine, Sahlgrenska Academy, University of Gothenburg, Gothenburg, Sweden

**Keywords:** Adolescent, adolescent psychiatry, child, child psychiatry, cross-sectional study, medical education, primary health care

## Abstract

**Introduction:**

Since the 1990s, mental ill-health among children and adolescents in Sweden has risen steadily, now ranking among the highest in Europe. Despite this, general practitioners’ and resident physicians’ education in primary healthcare in child and adolescent psychiatry is limited. Nevertheless, these professionals are expected to possess broad competence in managing mental ill-health in young patients. This study of general practitioners and resident physicians in primary healthcare regarding children and adolescents with mental ill-health aims to map (1) the self-assessed amount of education in child and adolescent psychiatry, (2) self-perceived competence in different types of symptoms, and 3) self-perceived competence when prescribing psychiatric medications.

**Methods:**

A cross-sectional study was conducted using web-based questionnaires completed by 184 general practitioners and 144 resident physicians in Sweden’s second-largest region. Data were analysed using Pearson’s Chi-squared test, Fisher’s exact test, Spearman’s correlation test, and the Holm–Bonferroni method.

**Results:**

Most participants reported limited education in child and adolescent psychiatry. Further, they reported lower competence in managing mental ill-health and prescribing drugs for children (6–12 years) compared to adolescents (13–17 years). Particularly low competence was reported in self-harm, eating disorders, and substance-related disorders.

**Conclusions:**

The growing prevalence of mental ill-health among children and adolescents is increasing pressure on primary care, with more young patients seeking help and higher expectations on providers. This study reveals that general practitioners and resident physicians often feel insufficiently prepared to manage these cases, highlighting gaps in medical training and emphasizing the need for strengthened medical school curricula, residency programs, and continuing medical education.

## Introduction

Mental ill-health among children and adolescents has been increasing steadily since the 1990s [1,2]. In accordance with the Swedish framework presented in Concepts within Mental Health [3], we use the term mental ill‑health, which differentiates between mental health problems and psychiatric conditions based on whether symptoms meet the criteria for a formal diagnosis. Many children and adolescents who present in primary care with concerns such as sleep difficulties or anxiety exhibit symptoms that do not reach diagnostic thresholds. Swedish children report among the highest levels of sleep difficulties and depression in Europe [4]. In 2023, 7.4% of children and adolescents up to age 17 in Sweden had contact with a child and adolescent psychiatric clinic [5]. However, there are no national statistics on how many children and adolescents seek primary healthcare for mental ill-health.

### Mental ill-health primary care organisation

In Region Västra Götaland, Sweden’s second-largest region, primary healthcare plays a central role in the initial assessment and management of mental ill-health in children and adolescents aged 6 to 17 [6]. Primary healthcare holds significant responsibility for the early identification and treatment of mild to moderate mental ill-health, including suicide risk evaluation and the provision of psychological interventions such as treatment for mild depression and anxiety [7]. When mental ill-health is more severe or when initial treatment proves ineffective, patients are referred to specialised child and adolescent psychiatric services. More complex or specialised care, such as in-depth psychological therapy, pharmacological treatment, or diagnostic assessments for conditions like attention-deficit/hyperactivity disorder (ADHD), autism spectrum disorder (ASD), bipolar disorder, and psychosis, is provided by child and adolescent psychiatry (CAP). To ensure that each patient receives care at the appropriate level, a structured model for consultation and collaboration between primary healthcare and CAP is in place [6]. In the latest agreement from 2025, more mental ill-health in young people, including mild difficulties with concentration, social interaction, and/or activity regulation, should be treated in primary healthcare [6]. However, the level of competence in primary healthcare to handle these diagnoses is not mapped.

Twenty-nine of the 210 healthcare centres in Region Västra Götaland have had an additional mission since November 2024: Youth People’s Mental Health (YPMH) [8]. This arrangement is unique to Sweden. These units are to offer psychoeducation, short-term treatment, internet-based cognitive behavioural therapy, and support for other agencies.

### Educational constraints

Traditionally, medical education in Sweden consists of a 5.5-year university programme at one of the seven universities with medical education. This is followed by an 18-month internship (AT), which is required to obtain a medical license. The internship includes a one-month voluntary placement, with the possibility to choose a placement at a CAP clinic. Specialist training in general medicine includes clinical work under supervision and different structured educational activities. It is mandatory to train in internal medicine, paediatrics, gynaecology, and adult psychiatry.

In 2021, a new structure was introduced that extended the university programme to six years and made it a licensure. The licensed physicians do not work as interns. Instead, they begin their specialist education with basic training (BT), a mandatory first part of the resident physicians’ training. BT includes no voluntary placement, so the opportunity to work at a CAP clinic disappears.

Approximately one-third of Swedish medical students receive their education abroad, often due to limited admission opportunities within Sweden [9]. Additionally, many physicians relocate to Sweden from other countries. For non-native speakers, language barriers, particularly in psychiatric consultations, can pose significant challenges [10].

Being a general practitioner is a multifaceted profession that requires broad medical expertise and the ability to manage various health problems. Although it takes at least 12 years to become a specialist in general medicine in Sweden, including medical education, internship, and a residency programme, the education in CAP is limited. There is no formal obligation to work at a CAP clinic or take a mandatory course during the internship and residency programme [11,12]. The Swedish Association for General Medicine, the specialist association, has no recommendations regarding education in child and adolescent psychiatric clinics. Competence in CAP, medical knowledge, and practical skills are expected to be developed through clinical experience at the primary healthcare centre.

On one hand, the number of children with mental ill-health is increasing in Sweden [4], and the responsibility for these patients in primary care is growing [6]. On the other hand, the short and varying scope of training in CAP during the medical programme and in the continuing medical education means that it is unclear whether the required medical competence in CAP at the primary care level is sufficient. Thus, there is a risk of developing a competence gap that results in a patient safety issue as well as a work environment issue. The latter is due to the increased stress on physicians in primary care, which can result from the discrepancy between the required medical skills and knowledge and their self-perceived competence [13].

This study aims to explore Swedish general practitioners’ and resident physicians’ self-perceived competence in managing mental ill-health among children and adolescents in primary healthcare. Specifically, it investigates three key areas: (1) their self-assessed amount of education in CAP, (2) their self-assessed competence in handling various mental ill-health, and (3) their confidence in prescribing psychotropic medications.

## Material and methods

### Settings

#### Organisational

Study participants were recruited only in Region Västra Götaland, sometimes described as Sweden in miniature. The region has a population of 1.8 million people and includes both the large city, Gothenburg, and rural areas. There are 210 healthcare centres in the region. Of these, approximately half are run publicly through Närhälsan, which has 106 healthcare centres evenly distributed throughout the region. There are also many smaller private chains operating healthcare centres in the region. Praktikertjänst is one of the largest private healthcare providers, operating 21 healthcare centres across Region Västra Götaland.

#### Youth people’s mental health units

Most YPMH units are located adjacent to private healthcare centres and eight to Närhälsans publicly run healthcare centres. A YPMH unit must be staffed with at least one licensed psychologist and one social worker. No physicians are employed by the YPMH unit; instead, they are employed by the healthcare centre where the unit is located. Families have the option to seek care at a YPMH unit even if they are not registered with the healthcare centre where the unit is located. A child treated at a YPMH unit sometimes changes healthcare centres to the one with the YPMH unit because contact with the physician becomes smoother. There are no formal regulations governing how children and adolescents should be allocated. At many healthcare centres, these patients are distributed among all physicians, while at others, physicians focus on different areas, some on child healthcare and others on residential care homes for the elderly or YPMH [14].

#### Child and adolescent psychiatry clinics

Region Västra Götaland hosts several CAP clinics distributed across the region, with referrals commonly coming from primary care or school health services. In 2022, the referral system was centralised to ‘One way in’ (*En väg in*, in Swedish) to streamline access and unify assessments. In 2023, primary care submitted 4,114 referrals, with 69% accepted [15], highlighting differing views between primary care physicians and the assessment team regarding treatment responsibility.

### Study participants and data collection

In this study, participation invitations were emailed to 127 healthcare centres, including all 800 physicians employed by the public primary healthcare provider Närhälsan and all 160 physicians employed by the privately run Praktikertjänst AB. It was not possible to identify which individuals had responded to the survey and which had not, and therefore, no targeted follow‑up reminders to non‑responders could be conducted. Many of the remaining 83 healthcare centres were affiliated with smaller and independent companies. Therefore, the study was also presented at some regional and local gatherings for resident physicians and general practitioners. Furthermore, the director of studies for the resident physicians and the healthcare centres’ managers were emailed about the study. Data collection took place between May and December 2024. Participants received a link to the digital survey.

### Questionnaire

A questionnaire was developed for the study (see Appendix). The survey items were reviewed for comprehensibility through pilot testing within the research group and with a small number of clinicians to ensure that the wording was clear and easy to interpret. The questionnaire was further refined for congruence through iterative feedback within the research team, and its content was primarily based on the care agreement between primary healthcare and CAP in Region Västra Götaland.

The survey began with an introductory section providing participants with information about the study’s purpose and procedures. This was followed by questions regarding the respondent’s gender identity and professional category. Subsequent items addressed whether the respondent had their medical education in Sweden and the self-assessed amount of education in CAP throughout different stages of their education. Participants were asked whether they worked at a healthcare centre with YPMH units.

They were then asked to rate their overall competence in independently managing children and adolescents with mental ill-health and were then divided into categories such as depression and sleep difficulties. The questionnaire included multiple-choice items (with 1–4 selectable options) and statements rated on a four-point Likert scale: ‘Very low competence’, ‘Low competence’, ‘Medium competence’, and ‘High competence’.

Additional questions explored the respondents’ perception of both their own and the healthcare centres’ overall competence in CAP. One question was about the perception of how the referral procedure to CAP *via* ‘One way in’ works.

Furthermore, the self-reported competencies in drug prescription were estimated with statements on a 10-point Likert scale. Finally, a question addressed the respondent’s own perceived competence in managing children and adolescents with somatic symptoms.

### Data analysis

Statistical analysis included both descriptive and analytical statistics. The number of BT physicians in the study is very low; therefore, they have been included in the group of resident physicians, as their training is an initial part of specialist education.

Pearson’s chi-squared test, Fisher’s exact test, Spearman’s correlation test, and the Holm–Bonferroni method were applied in the analyses. All statistical analyses were performed using the IBM Statistical Package for the Social Sciences, SPSS 29 statistical package for Windows (IBM Corp., Armonk, NY).

## Results

The study included 184 general practitioners and 144 resident physicians in primary care. More women (*n* = 180) than men responded to the survey; the majority had studied medicine in Sweden (*n* = 216; data not shown). Most respondents worked in the subregion of Skaraborg (*n* = 112), one of the five administrative regions in Region Västra Götaland, with approximately 250,000 inhabitants. Almost half of the 116 physicians working at a healthcare centre with a YPMH unit responded to the survey (*n* = 56).

**Figure 1.**
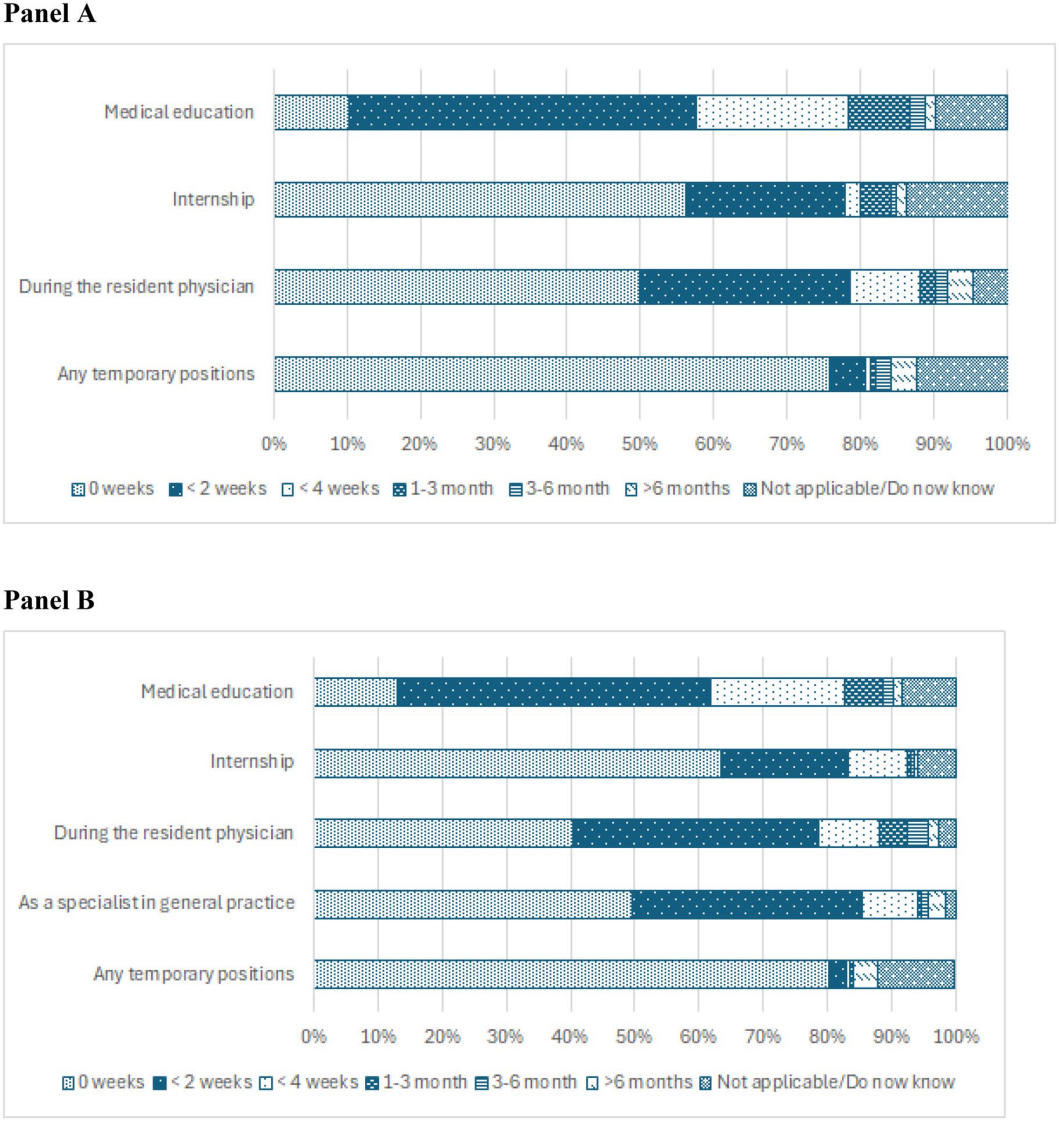
Panel A: Amount of self-assessed education in weeks in child and adolescent psychiatry for resident physicians (ST) in general medicine. Panel B: The amount of self-assessed education in weeks in child and adolescent psychiatry for the general practitioners.

A clear majority of resident physicians in general medicine self-assessed that they have received little (less than 2 weeks) to no education in CAP (Figure 1, Panel A). Similarly, general practitioners typically self-assessed that they had no or only a few days to less than two weeks of education, throughout their career stages (Figure 1, Panel B). Less than 5% of the responding general practitioners held temporary positions lasting more than six months within CAP.

**Figure 2.**
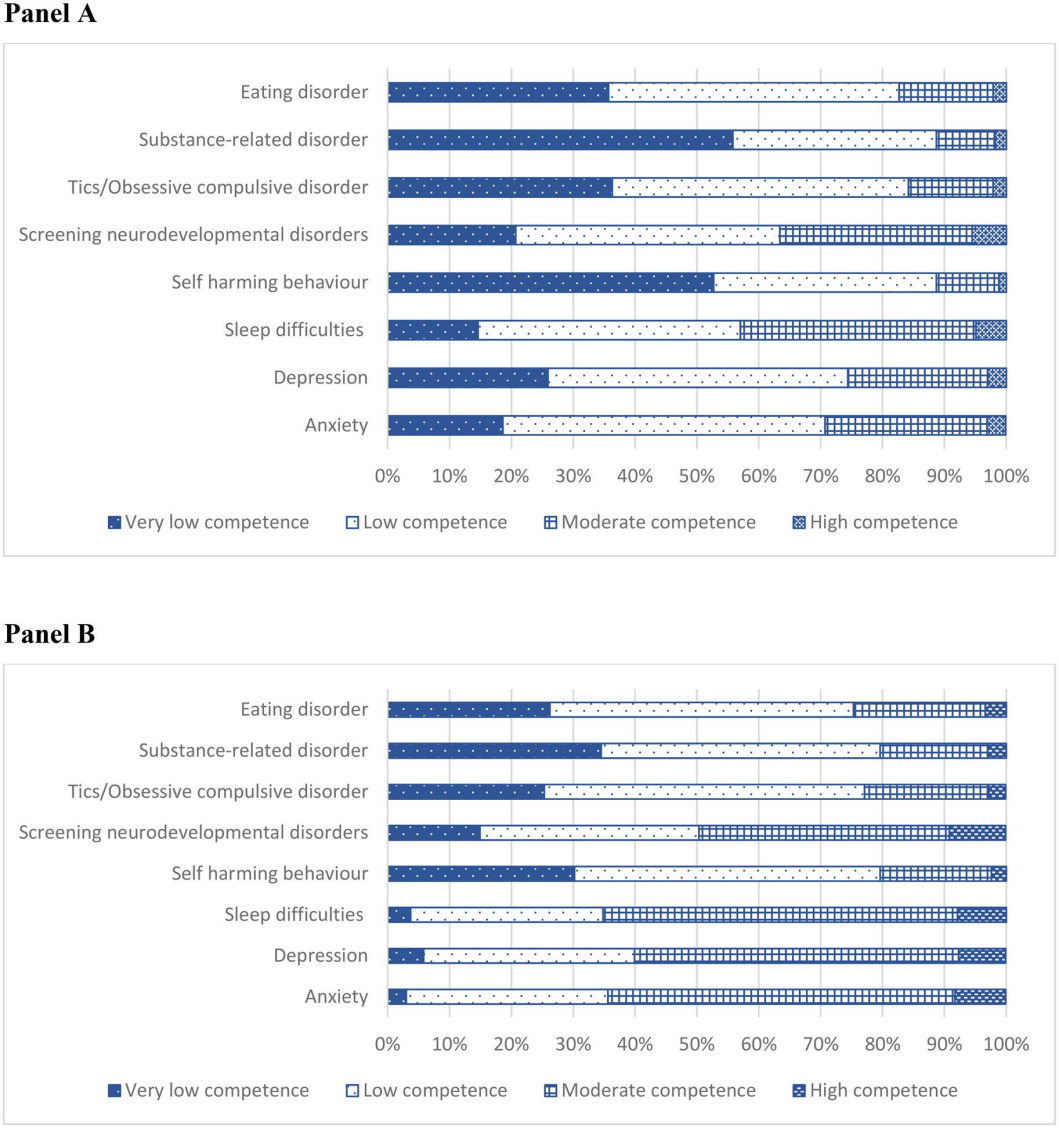
Panel A: Self-perceived competence of all physicians in independently managing children aged 6–12 with specific symptoms of a mild to moderate nature. Panel B: Self-perceived competence in independently managing adolescents aged 13–17 years with specific symptoms of a mild to moderate nature.

The self-perceived competence in managing children with mental ill-health is generally low (Figure 2, Panel A). Self-perceived competence was generally higher for symptoms such as anxiety, depression, sleep difficulties, and screening for neurodevelopmental disorders such as ASD and ADHD. In contrast, confidence was notably lower regarding self-harming behaviour, eating disorders, tics/obsessive-compulsive disorder, and substance-related disorders. The self-perceived competence in managing adolescents is higher compared with children (Figure 2, Panel B). A clear majority of surveyed physicians, between 80% and 90%, expressed a desire for further education in CAP regarding all the symptoms included in the survey.

The perception of how the referral procedure to CAP functions *via* ‘One way in’ was assessed using statements rated on a Likert scale ranging from 0 (‘It does not work at all’) to 10 (‘It works very well’). The results indicated a low average rating of 3.0 (± 2.1) on the 10-point scale (data not shown).

Drug prescription was estimated with statements on a Likert scale ranging from 0 (‘Very unsafe’) to 10 (‘Completely safe’). Many physicians feel more confident prescribing psychotropic drugs to adolescents than to children. The average confidence level for prescribing psychotropic drugs to children aged 6–12 years was 2.5 (±2.1), while the average for adolescents aged 13–17 years was 4.2 (±2.2). The difference between the groups was statistically significant (*p* < 0.0001).

In contrast to mental ill-health, self-perceived competence in managing somatic symptoms in children and adolescents was generally high. Specifically, 67% rated ‘High competence’ and 29% ‘Medium competence’.

### Youth people’s mental health

To investigate potential differences in self-perceived competence between physicians working at healthcare centres with a YPMH unit (*n* = 56) and those at other healthcare centres (*n* = 272), responses regarding both individual and overall competence in independently managing children and adolescents with mental ill-health were analysed.

When comparing the overall competence at the healthcare centre, a clear difference emerged: physicians at centres with a YPMH unit rated their overall competence higher (*M* = 2.8, SD = 0.7) than those at centres without a YPMH unit (*M* = 2.3, SD = 0.7; *p* < 0.0001).

## Discussion

### Main findings

The study shows that both general practitioners and resident physicians in primary healthcare self-assessed a limited education in CAP, often none or only a few weeks. The self-perceived competence in independent treatment and prescribing of medication is lower for children than for adolescents with mental ill-health. Considering the increasing rates of mental ill-health among children and adolescents and the growing expectation that more of these mental health problems and psychiatric conditions should be treated in primary healthcare, there is a clear need for strengthened medical school curricula, residency programs, and continuing medical education.

## Discussion of the results and comparison with existing literature

### Education in CAP is in discrepancy with the responsibility of primary care physicians

Accurately assessing the extent of university-level education in CAP can be difficult for physicians, especially when the training occurred many years ago. Differences in curriculum length and content between institutions and across time further complicate such evaluations. A German study found that the CAP-related knowledge and skills perceived as relevant by primary healthcare physicians differ significantly from those emphasised in most current medical school curricula [16]. In Sweden, a 2010 report from the Swedish National Audit Office demonstrated that the representation of CAP and general psychiatry in medical education had declined during the first decade of the 2000s, mirroring findings reported in Germany [17].

Similarly, a Nordic study reported that physicians felt insufficiently competent in several areas of CAP [18]. Although clinical experience likely contributes to skill development, our findings indicate that it does not fully compensate for the lack of self-perceived competence in CAP [19]. CAP education should be updated to better align with the needs of general practitioners, thereby improving care for children and adolescents with mental ill-health through strengthened medical school curricula, residency programs, and continuing medical education [20].

Increased CAP training and education are more urgent than ever for primary healthcare physicians, as more mental ill-health in young people in Region Västra Götaland should be treated in primary healthcare according to the agreement between primary healthcare and CAP [6]. Based on this study’s results, expanded CAP training, a mandatory CAP course during residency and continuing medical education for primary care physicians must precede these decisions on increased responsibility for children and adolescents with mental ill-health at primary healthcare to provide safe care for the patients and improve physicians’ feelings of their own confidence in managing the mental ill-health in children and adolescents. Specifically, regarding younger children, as this study pointed out, the observed differences in competencies were based on child age groups. These differences may partly be explained by the fact that mental ill-health in children sometimes manifest differently than in adults, frequently presenting as somatic complaints [21,22], and that adolescents are more similar to adults in symptoms and treatment.

In contrast to CAP, self-perceived competence in managing somatic symptoms in children and adolescents is generally high [23]. This may partly be due to the mandatory paediatric rotation during general medicine specialist training. At most primary care centres, general practitioners and resident physicians regularly encounter children with various somatic complaints. Training and hands-on experience foster knowledge and confidence in treating these patients.

### Digital consultation as a response to the primary care physician’s need for advice

General practitioners in this study report low confidence in managing mental ill-health in children and adolescents. This lack of competence may contribute to uncertainty in clinical decision-making and increase the physician’s strain. The situation is further exacerbated when referrals to ‘One way in’ are rejected, despite the physician’s assessment of the need for specialist psychiatric care. Such discrepancies between primary care and CAP referral assessments might lead to feelings of professional inadequacy and stress, especially when physicians in primary healthcare who are responsible for the patient believe they require care beyond their expertise. These challenges underscore the importance of lowering the threshold between primary and specialist care by fostering closer collaboration. Rather than working in parallel silos, integrated efforts are needed to ensure timely and appropriate care for children and adolescents with mental ill-health. Creating opportunities such as digital consultations between primary care physicians and CAP specialists might be the right next step. Thus, in some cases, primary care physicians can receive help in managing mental ill-health without needing to refer patients to the ‘One way in’ process, saving time and effort. These digital consultation possibilities could also serve as educational opportunities for physicians.

### Competencies in the prescription of psychotropic medications

Our Swedish study indicates a generally low level of self-perceived competence among general practitioners in both the management and prescription of psychotropic medications for children and adolescents. This finding is consistent with previous research from the United Kingdom, the United States, and Norway [19,23,24].

The low self-perceived competence in prescribing psychotropic medications, particularly for children, and to a lesser extent for adolescents, has also been reported in Dutch primary care settings [25]. Comparative data between Sweden and Denmark show that initial prescriptions of antidepressants are issued to a much lesser extent in Swedish primary care than in Denmark. This may be related to Sweden’s relatively higher access to child and adolescent psychiatric services compared to Denmark and Norway [20].

The issue of prescribing psychotropic medication in primary care is particularly relevant in the light of the high prevalence of ADHD in Sweden. In May 2024, the Swedish Ministry of Health and Welfare commissioned the Medical Products Agency to increase knowledge about the use of ADHD medications. The assignment includes a review of current treatment guidelines and assessing whether prescribing rights could be extended to more physicians outside of psychiatric care [26]. Should general practitioners be given increased responsibility for prescribing these ADHD medications, it may place additional strain on primary care services. Our findings suggest that many physicians already experience limited competence and uncertainty in treating children and adolescents with psychotropic medications, which is confirmed in other studies [27,28]. This further underscores that strengthened medical school curricula, residency programs, and continuing medical education in CAP must come before implementing decisions that expand the responsibilities of primary care physicians.

### Youth people’s mental health

Physicians working at healthcare centres with an affiliated YPMH unit rated their competence significantly higher compared with physicians at healthcare centres without such a unit. While it is reasonable to assume that these physicians encounter more children and adolescents with mental ill-health, the observed effect is modest, and the number of physicians in these centres remains limited. The YPMH unit offers conversational therapy to young patients, who can continue their contact with their regular physician at the healthcare centre. It is common for patients to register with a centre that includes a YPMH unit to facilitate collaboration between the physician and the unit. This arrangement may provide physicians with more hands-on experience, thereby increasing their confidence in independently managing children and adolescents with mental ill-health [29].

Interviews with resident physicians provide insight into the importance of a clear curriculum, supervision, and a combination of practical experience and reflection [30]. It can be challenging to supervise a general practitioner [31]; further, it can be challenging to teach them CAP if they feel insecure, have inadequate training, and rarely encounter children and adolescents with CAP issues. As a complement to a CAP clinic service, a period of service at a healthcare centre with a YPMH unit and a committed, skilled physician knowledgeable in CAP would be of value for all resident physicians. Student-run clinics in primary healthcare have earlier shown a great potential for student-regulated learning [32].

To better address mental ill-health among children and adolescents, it would be valuable to review CAP training in Sweden for resident physicians in primary healthcare. In addition, further investigation is needed into what measures could enhance general practitioners’ self-perceived competence in managing children and adolescents with mental ill-health and improve confidence when prescribing psychotropic medication. Additional educational initiatives appear highly relevant and have been proven effective in primary care [33,34]. Given the rising prevalence of mental ill-health among children and young people, resident physicians and general practitioners must possess adequate knowledge and skills to appropriately meet the needs of this patient group.

### Strengths and weaknesses of the study

The use of anonymous survey responses likely increased the honesty and reliability of the data, strengthening the overall quality. It can be embarrassing for an experienced general practitioner to admit that they feel they lack knowledge. By comparing self-perceived competence among physicians working in healthcare centres with and without YPMH units, the study offers valuable insights into how new organisational structures may influence the clinic.

Due to practical limitations, it was not possible to reach all physicians directly *via* email. Physicians affiliated with Närhälsan and Praktikertjänst were contacted electronically, while some of the others were reached indirectly through healthcare centre managers, the director of studies for resident physicians, and during local gatherings for primary healthcare physicians. It may be that the healthcare centre managers and the director of studies for resident physicians did not forward the email survey to the physicians. Since the researchers conducting the study are general practitioners based in Skaraborg, a higher proportion of participants came from that subregion.

It is impossible to identify those who did not participate in the survey, as the respondents were anonymous. Some physicians, especially resident physicians, were on parental leave or side assignments at other clinics during the data collection and, therefore, did not respond to the survey.

A large proportion of the physicians at healthcare centres with YPMH units responded to the survey. While the exact respondent profile is unknown, it is likely that those who completed the survey were more engaged, motivated, and had the time to participate. Therefore, there is a risk of selection bias. It should also be noted that the survey’s design allowed for the possibility of multiple responses from the same individual. Despite these limitations, we consider the sample to be representative for this study.

It is important to acknowledge that this study assesses only self-perceived competence, which may not reflect the actual clinical competence of individual general practitioners. Previous research has demonstrated that physicians’ self‑assessed competence does not always align with observed clinical performance. Several studies indicate that self‑assessment is often an unreliable indicator of clinical skill, with physicians, particularly those with lower objective performance, tending to overestimate their abilities [34,35]. Although some studies have reported modest or context‑dependent associations between confidence and observed competence [36], the overall evidence suggests that self‑perceived competence should be interpreted with caution. These findings underscore the need for future research to examine how primary care physicians’ self‑assessed readiness to manage child and adolescent mental ill-health corresponds to their actual clinical skills and training needs. Therefore, the results should not be interpreted as a definitive measure of the true level of CAP-related knowledge within the general medical profession. Nevertheless, similar findings have been reported in other studies, where general practitioners have estimated their CAP competence to be relatively low [16,37].

## Conclusions

The prevalence of mental ill-health among children and adolescents is increasing, and there is a growing expectation that primary healthcare should manage a larger share of mental ill-health in this population. However, the current structure of medical training and education offers limited and inconsistent exposure to CAP, raising concerns about whether primary care physicians possess the necessary competence to meet these demands.

Our study confirms that both resident physicians and general practitioners report low self-perceived competence in managing mental ill-health, particularly in children. This perception reflects a significant lack in both undergraduate, residency period, and continuing medical education related to CAP.

These findings underscore the urgent need for structured and continuous educational initiatives aimed at strengthening competence in CAP within the primary care sector. Without such efforts, the mismatch between clinical expectations and physician preparedness may pose risks to patient safety and contribute to increased occupational stress among healthcare providers.

## Supplementary Material

Appendix Caring for Young Minds.docx
