# Supplementary material for: Caring for young minds: general practitioners’ self-assessed competence in child and adolescent psychiatry
Source: Scand J Prim Health Care. 2026 Apr 15;44(1):2654175. doi: 10.1080/02813432.2026.2654175 (PMC13084840; doi:10.1080/02813432.2026.2654175)
Supplement: Appendix Caring for Young Minds.docx [file IPRI_A_2654175_SM4636.docx]

**Appendix**

**Survey**

1. Which gender do you identify with? *Select one option only.*

- Female
- Male
- Other

2. Did you complete your medical degree in Sweden? *Select one option only.*

- Yes
- No

3. In which area of Region Västra Götaland do you currently work? *Select one option only.*

- Skaraborg
- Södra Bohuslän
- Södra Älvsborg
- Göteborg
- Fyrbodal

4. What is your current professional role?

- Resident physician
- Specialist in general practice
- Basic training physician

5. How much total training—comprising theoretical education, clinical practice, and external clinical placements—related to child and adolescent mental health have you received during each stage of your medical training or employment?

*This question applies to resident physicians and basic training physicians only.*

|  | 0 week | <2 weeks | 2–4 weeks | 1–3 months | 3–6 months | Not applicable /Do not know |
| --- | --- | --- | --- | --- | --- | --- |
| Medical education | o | o | o | o | o | o |
| Internship | o | o | o | o | o | o |
| Residency programme | o | o | o | o | o | o |
| Any temporary positions | o | o | o | o | o | o |

6. How much total training—including theoretical education, clinical practice, and external clinical placements—related to child and adolescent mental health have you received during each of the following stages of your medical career?

*This question applies to specialists in general practice.*

|  | 0 week | <2 weeks | 2–4 weeks | 1–3 months | 3–6 months | Not applicable/Do not know |
| --- | --- | --- | --- | --- | --- | --- |
| Medical education | o | o | o | o | o | o |
| Internship | o | o | o | o | o | o |
| Residency programme | o | o | o | o | o | o |
| As a specialist in general practice | o | o | o | o | o | o |
| Any temporary positions | o | o | o | o | o | o |

7. Do you work at a primary care center with the additional assignment ‘Youth people's Mental Health’ – YPMH?

- Yes
- No

8. How do you rate your own self-perceived competence in independently managing children aged 6–12 years presenting with the following symptoms of mild to moderate severity?

1. = Very low competence
2. = Low competence
3. = Moderate competence
4. = High competence

|  | 1 | 2 | 3 | 4 |
| --- | --- | --- | --- | --- |
| Anxiety | o | o | o | o |
| Depression | o | o | o | o |
| Sleep disorder | o | o | o | o |
| Self-harming behaviour | o | o | o | o |
| Screening for neuropsychiatric diagnosis | o | o | o | o |
| Tics / Obsessive-compulsive disorder | o | o | o | o |
| Substance use disorder / Addiction | o | o | o | o |
| Eating disorder | o | o | o | o |

9. How do you rate your overall self-perceived competence in independently managing children aged 6–12 years with mental health problems (including all types of symptoms)?

1. = Very low competence
2. = Low competence
3. = Moderate competence
4. = High competence

|  | 1 | 2 | 3 | 4 |
| --- | --- | --- | --- | --- |
| Overall competence | o | o | o | o |

10. How confident do you feel in your medical competence when prescribing psychotropic medications (antidepressants, sleep aids, and anxiolytics) to children aged 6–12 years with mental health problems?

| 0  Very unconfident | 1 | 2 | 3 | 4 | 5 | 6 | 7 | 8 | 9 | 10  completely confident |
| --- | --- | --- | --- | --- | --- | --- | --- | --- | --- | --- |
| o | o | o | o | o | o | o | o | o | o | o |

11. How do you rate your own self-perceived competence in independently managing adolescents aged 13–17 years presenting with the following symptoms of mild to moderate severity?

1. = Very low competence
2. = Low competence
3. = Moderate competence
4. = High competence

|  | 1 | 2 | 3 | 4 |
| --- | --- | --- | --- | --- |
| Anxiety | o | o | o | o |
| Depression | o | o | o | o |
| Sleep disorder | o | o | o | o |
| Self-harming behaviour | o | o | o | o |
| Screening for suspected neuropsychiatric diagnosis | o | o | o | o |
| Tics / Obsessive-compulsive disorder | o | o | o | o |
| Substance use disorder / Addiction | o | o | o | o |
| Eating disorder | o | o | o | o |

12. How do you rate your overall self-perceived competence in independently managing adolescents aged 13–17 years with mental health problems (including all types of symptoms)?

1. = Very low competence
2. = Low competence
3. = Moderate competence
4. = High competence

|  | 1 | 2 | 3 | 4 |
| --- | --- | --- | --- | --- |
| Overall competence | o | o | o | o |

13. How confident do you feel in your self-perceived medical competence when prescribing psychotropic medications (antidepressants, sleep aids, and anxiolytics) to adolescents aged 13–17 years with mental health problems?

| 0  very unconfident | 1 | 2 | 3 | 4 | 5 | 6 | 7 | 8 | 9 | 10  completely confident |
| --- | --- | --- | --- | --- | --- | --- | --- | --- | --- | --- |
| o | o | o | o | o | o | o | o | o | o | o |

14. Are you familiar with the division of responsibilities between primary care and child and adolescent psychiatry?

- Yes
- No

15. Do you apply the division of responsibilities between child and adolescent psychiatry and primary care when managing children and adolescents with mental health problems?

- Yes
- No

16. How do you perceive the referral process to child and adolescent psychiatry (‘One way in’)?

*In 2022, the referral system to child and adolescent psychiatry was reorganized into a region-wide referral center in Gothenburg called ‘One Entry Point’. Since then, all referrals from primary care in Region Västra Götaland are sent to this centralized referral unit for assessment.*

| 0  It does not work at all | 1 | 2 | 3 | 4 | 5 | 6 | 7 | 8 | 9 | 10  It works very well |
| --- | --- | --- | --- | --- | --- | --- | --- | --- | --- | --- |
| o | o | o | o | o | o | o | o | o | o | o |

17. How do you perceive your primary care centre’s overall competence—including all professional categories (physicians, nurses, assistant nurses, and possibly therapists and psychologists)—in managing children and adolescents with mental health problems?

1. = Very low competence
2. = Low competence
3. = Moderate competence
4. = High competence

|  | 1 | 2 | 3 | 4 |
| --- | --- | --- | --- | --- |
| Overall competence | o | o | o | o |

18. How do you perceive your own competence in examining and managing children and adolescents with somatic symptoms, such as infection symptoms, asthma, and constipation?

1. = Very low competence
2. = Low competence
3. = Moderate competence
4. = High competence

|  | 1 | 2 | 3 | 4 |
| --- | --- | --- | --- | --- |
| Overall competence | o | o | o | o |

19. In which of the following areas within child and adolescent psychiatry would you like to receive more training?

|  | No additional training needed | More training desired |
| --- | --- | --- |
| Anxiety | o | o |
| Depression | o | o |
| Sleep disorder | o | o |
| Self-harming behaviour | o | o |
| Screening for suspected neuropsychiatric diagnosis | o | o |
| Tics / Obsessive-compulsive disorder | o | o |
| Substance use disorder / Addiction | o | o |
| Eating disorder | o | o |
